# Cellular localization and trafficking of vascular adhesion protein-1 as revealed by an N-terminal GFP fusion protein

**DOI:** 10.1007/s00702-013-1003-3

**Published:** 2013-03-09

**Authors:** Chris J. Weston, Emma L. Shepherd, David H. Adams

**Affiliations:** Centre for Liver Research and NIHR Biomedical Research Unit, 5th Floor Institute of Biomedical Research, MRC Centre for Immune Regulation, College of Medicine and Dentistry, University of Birmingham, Edgbaston, Birmingham, B15 2TT UK

**Keywords:** Vascular adhesion protein-1, Amine oxidase, Trafficking, Imaging, Liver

## Abstract

Recent studies of vascular adhesion protein-1 (VAP-1) have greatly advanced our understanding of the important role this protein plays in the establishment and progression of inflammatory disease. To facilitate more detailed studies on the function of VAP-1, we developed a GFP-fusion protein that enabled us to monitor the trafficking of the protein in three selected cell types: hepatic sinusoidal endothelial cells, liver myofibroblasts and an hepatic stellate cell line (LX-2). The fusion protein was detected as punctate cytoplasmic GFP staining, but was present only at low levels at the cell surface in all cell types studied. The subcellular distribution of the protein was not altered in a catalytically inactive mutant form of the protein (Tyr471Phe) or in the presence of exogenous VAP-1 substrate (methylamine) or inhibitor (semicarbazide). The GFP-VAP-1 protein was localized to the Golgi apparatus (GM-130), endoplasmic reticulum (GRP94) and early endosomes (EEA-1). Additional staining for VAP-1 revealed that the overexpressed protein was also present in vesicles that were negative for GFP fluorescent signal and did not express EEA-1. We propose that these vesicles are responsible for recycling the fusion protein and that the fluorescence of the GFP moiety is quenched at the low pH within these vesicles. This feature of the protein makes it well suited for live cell imaging studies where we wish to track protein that is being actively trafficked within the cell in preference to that which is being recycled.

**Electronic supplementary material:**

The online version of this article (doi:10.1007/s00702-013-1003-3) contains supplementary material, which is available to authorized users.

## Introduction

Vascular adhesion protein-1 (VAP-1, AOC3) is a transmembrane protein that is expressed in endothelial, adipose and smooth muscle cells and has been shown to support the recruitment of immune cells into sites of inflammation through interactions with ligands, such as Siglec-9/Siglec-10 or via deamination of primary amines (for recent reviews see (Weston and Adams 2011; Salmi and Jalkanen 2011)).

The expression of VAP-1 in primary hepatic sinusoidal endothelial cells is not altered upon treatment with common inflammatory mediators (Lalor et al. 2002) and several groups have studied the expression of VAP-1 in cells that have been modified to overexpress the protein on their surface. The recruitment of immune cells was enhanced in cells overexpressing VAP-1 via a mechanism that was, in part, dependent on the enzymatic action of VAP-1 (Koskinen et al. 2004; Salmi et al. 2000).

Until recently, little was known about the cellular distribution of VAP-1 except that it can be mobilised from intracellular stores and trafficked to the membrane during inflammation (Salmi and Jalkanen 2001). Sole et al. updated this model by creating both an endothelial cell line from human umbilical vein endothelial cells (HUVEC) and a smooth muscle cell line (A7r5) that expressed VAP-1 at high levels (Sole and Unzeta 2011), where the protein was observed to associate with lipid rafts and Golgi/endosomal fractions.

To extend our knowledge of the trafficking of VAP-1, we expressed a GFP-fusion protein in both endothelial and stromal cells and used multicolour confocal microscopy to determine the cellular distribution of VAP-1 and identify compounds that might affect how the protein is transported within the cell.

## Materials and methods

### Human tissue

Human tissue was obtained from the Queen Elizabeth Hospital (Birmingham, UK) after informed consent and with local ethics committee approval. Diseased liver tissue was taken from patients undergoing liver transplantation for chronic liver disease (CLD) and normal liver from uninvolved tissue removed at surgical resection for hepatic colorectal metastases or from surplus donor liver.

### Isolation of human activated liver myofibroblasts (aLMF) and HSEC

aLMF were purified from fibrotic tissue as described previously (Holt et al. 2009). Cells were plated on plastic in DMEM/16 % FCS (Invitrogen, UK) and used within four passages. Liver endothelial cells (HSEC) were isolated from human liver tissue obtained from explanted livers or donor tissue surplus to surgical requirements using a collagenase digestion and CD31-positive selection as described previously (Lalor et al. 2002). Cells were cultured in endothelial basal media (Invitrogen, UK) containing 10 % heat inactivated AB human serum (HD Supplies, Glasgow, UK), 10 ng/mL vascular endothelial growth factor (VEGF) and 10 ng/mL hepatocyte growth factor (HGF) (PeproTech, UK) in rat tail collagen-coated tissue culture flasks.

### Cell lines

LX-2, a low serum adapted HSC cell line (Xu et al. 2005), a kind gift from Scott Friedman (Mount Sinai, New York), were grown in DMEM supplemented with 2 % FCS. The human embryonic kidney cell line, HEK293 were obtained from the European Collection of Cell Cultures (ECACC) and were cultured in DMEM supplemented with 10 % FCS and 25 mM HEPES.

### Construction of plasmids encoding GFP-fusion proteins

The sequence for VAP-1 was sub-cloned out of a pcDNA3.1 vector containing the full-length VAP-1 sequence with a GFP tag at the 3′-end (wtVAP-1-GFP, a kind gift from David Smith, Biotie Therapies, Finland) into the pcDNA6.2N-EmGFP TOPO vector (Life Technologies) using primers coding for Asn2–Asn763 of the VAP-1 sequence followed by three stop codons (TAG TGA TAA) to reduce the frequency of read-through errors. The plasmid also codes for ampicillin and blasticidin selection, drives expression of the target protein from a CMV promoter region and provides an 18 amino acid linker region to connect the GFP moiety to the VAP-1 sequence (GSS PST SLYK KAG SEFAL). Forward primer: 5′-AACCAGAAGA CAATCCTCGT GCTCCTC-3′; reverse primer: 5′-TTATCACT AATTGTGAGA GAAGCCCCCG-3′. The orientation and correctness of the insert was confirmed by sequencing (Functional Genomics Laboratory, University of Birmingham). A mutation corresponding to a change in Tyr to Phe at amino acid position 471 (Y471F) which has been shown to prevent formation of the active site topaquinone was introduced using the QuikChange Site Directed Mutagenesis Kit (Stratagene, UK) and the following primers: forward 5′-ATGTCCACCT TGCTCAACTT CGACTATGTG TGGGATACGG-3′; reverse 5′-CCGTATCCCA CACATAGTCG AAGTTGAGCA AGGTGGACAT-3′. The identity of the mutant was verified by sequencing and is henceforth referred to as GFP-(Y471F)VAP-1. A pcDNA6.2N-EmGFP TOPO plasmid containing the sequence for the cytoplasmic protein chloramphenicol acetyl transferase (CAT) was used as an expression control (Invitrogen, UK).

### Transfection of cells

Cells were transiently transfected with plasmid DNA using a Nucleofector II (Lonza, UK) according to the manufacturer’s instructions. In brief, 2–10 μg DNA were Nucleofected into 0.5–1.0 × 10^6^ cells in 100 μl Nucleofection solution (basic primary endothelial cell kit for HSEC, program T-005; basic primary fibroblast kit for aLMF and LX-2, program A-023; nucleofector solution V, program Q-001 for HEK293) and transferred into tissue culture vessels containing the appropriate media.

### Treatment of transfectants with modulators of cellular trafficking and enzyme function

Cells (0.5 × 10^6^) were Nucleofected with 5 μg DNA, resuspended in 5 mL media and then aliquotted into each well of an Ibidi Slide 12 chamber slide (200 μL/well). The cultures were incubated at 37 C/5 % CO_2_ overnight after which the media was replaced with a low-serum-containing equivalent (endothelial basal media plus 2 % FCS for HSEC, DMEM containing 2 % FCS for aLMF and LX-2). At 48 h post-Nucleofection, selected wells were dosed with the following compounds in appropriate media and left to incubate for 16 h: (a) marimastat, 10 μM (Calbiochem, UK); (b) bafilomycin A1, 1 μM (Calbiochem, UK); (c) GolgiPlug (Brefeldin A), 1 μL/mL (BD Biosciences, UK); (d) Golgistop (Monensin), 0.7 μL/mL (BD Biosciences, UK). The cells were then fixed with 4 % paraformaldehyde for 10 min and stored in PBS at 4 C before being imaged as described above. For live cell staining, the samples were incubated for 30 min with control antibody (mouse IgG2a, Dako) or anti-VAP-1 antibody (TK8-14, BioTie Therapies) at 10 μg/mL prior to fixation as above. Separate wells were dosed at 72 h post-Nucleofection for 30 min with one of the following compounds: (a) methylamine, 1 mM (Sigma, UK); (b) semicarbazide, 250 μM (Sigma, UK); (c) cytochalasin D, 10 μM (Calbiochem, UK); (d) jasplakinolide, 500 nM (Calbiochem, UK); (e) nocodazole, 10 μM (Calbiochem, UK). The cells were then fixed with PFA as described above. For washout studies, wells were dosed with nocodazole for 30 min at which point the media was changed and the cells were fixed immediately with 4 % PFA or following a further incubation of 15 or 30 min in the absence of the inhibitor. In all cases, appropriate vehicle controls (DMSO or ethanol) were monitored in parallel.

### Amine oxidase assay

VAP-1 is a semicarbazide-sensitive amine oxidase (SSAO). Amine oxidase activity of live cells (80,000 cells per well in a 24 well plate) was measured using a continuous Amplex UltraRed based assay (Molecular Probes, Invitrogen, UK) according to the manufacturer’s instructions. Each reaction mix contained monoamine oxidase A and B inhibitors (clorgyline, pargyline, 200 μM each) and the artificial VAP-1 substrate benzylamine (1 mM) in the absence and presence of the SSAO inhibitor 2-bromoethylamine (200 μM, Sigma).

### Fluorescent staining of fixed cells

Fixed cells were permeabilised with 0.3 % Triton-X100 in PBS and subsequently blocked with 10 % goat serum in PBS containing 0.1 % Triton-X100. Primary antibodies raised against specific organelles (mouse-anti-GM130, mouse-anti-EEA1 from BD Biosciences both at 5 μg/mL; rabbit-anti-GRP94, 1/100, AbCAM; mouse-anti-LAMP-1, 5 μg/mL, BioLegend), rabbit-anti-GFP (D5.1, Cell Signaling, 1/50) and/or anti-VAP-1 antibody TK8-14 (mouse-anti-VAP-1, 10 μg/mL, BioTie Therapies) were diluted in PBS containing 0.1 % Triton-X100 and 2 % goat serum and added to the cells, and the samples were incubated at 4 C overnight. The cells were washed with PBS/0.1 % Triton-X100 and incubated with an appropriate Alexa Fluor conjugated secondary antibody in the same buffer for 1 h at room temperature (1:500 dilution of secondary, Alexa Fluor-546/Alexa Fluor-633, Invitrogen, UK). The wells were then washed with PBS/0.1 % Triton-X100, stained with DAPI and then mounted with Mowiol. Images were acquired using a Zeiss LSM 510 confocal fluorescence microscope (Carl Zeiss, Germany).

### Western blot

Total cell protein was isolated using CelLytic M lysis buffer (Sigma, UK) supplemented with complete ULTRA protease inhibitors, PhosSTOP phosphatase inhibitors and DNase I (all Roche Diagnostics, UK) according to the manufacturer’s instructions. Protein concentration was determined by bicinchoninic acid assay with BSA as standard (Sigma, UK). Samples (40 μg/lane for HEK293 and LX-2 lysates, 8 μg/lane for HSEC and aLMF lysates) were separated on 4 % SDS-PAGE gels, transferred on to nitrocellulose which were then blocked with 5 % milk powder in PBS containing 0.05 % Tween-20 (blotting buffer). The membranes were then incubated with primary antibody (TK8-14, 2 μg/mL, in blotting buffer) overnight at 4 C. Proteins were visualised using HRP-conjugated secondary antibodies (Rabbit-anti-mouse-HRP, 1:2,500 in blotting buffer, 1 h incubation at room temperature; Dako, UK) and SuperSignal West Pico chemiluminescent substrate (Pierce, UK). Recombinant VAP-1 (rVAP-1) produced in CHO, corresponding to the extracellular domain of VAP-1 (a kind gift from David Smith, Biotie Therapies, Finland), was used as a positive control.

## Results

### A VAP-1 GFP-fusion protein expressed by HEK293 was enzymatically active

VAP-1 is predicted to be a type II membrane protein with a short cytoplasmic tail of four amino acids (NQKT) with no known signal sequence, and a large extracellular domain which contains the enzyme active site. To better understand the cellular distribution of VAP-1, we designed a GFP fusion protein driven from a CMV promoter that incorporated an 18 amino acid linker connecting the GFP moiety to the N-terminus of VAP-1 (GFP-wtVAP-1). We designed a second expression vector in which the tyrosine at position 471 in the VAP-1 sequence was replaced by phenylalanine (GFP-(Y471F)VAP-1). This mutation prevents the formation of a topaquinone in the active site and has been shown to render the enzyme incapable of catalysis (Jalkanen et al. 2007). We also included a control plasmid-encoding GFP-tagged chloramphenicol acetyl transferase, a cytoplasmic protein, driven from the same CMV promoter (GFP-CAT, Invitrogen).

**Fig. 1:**
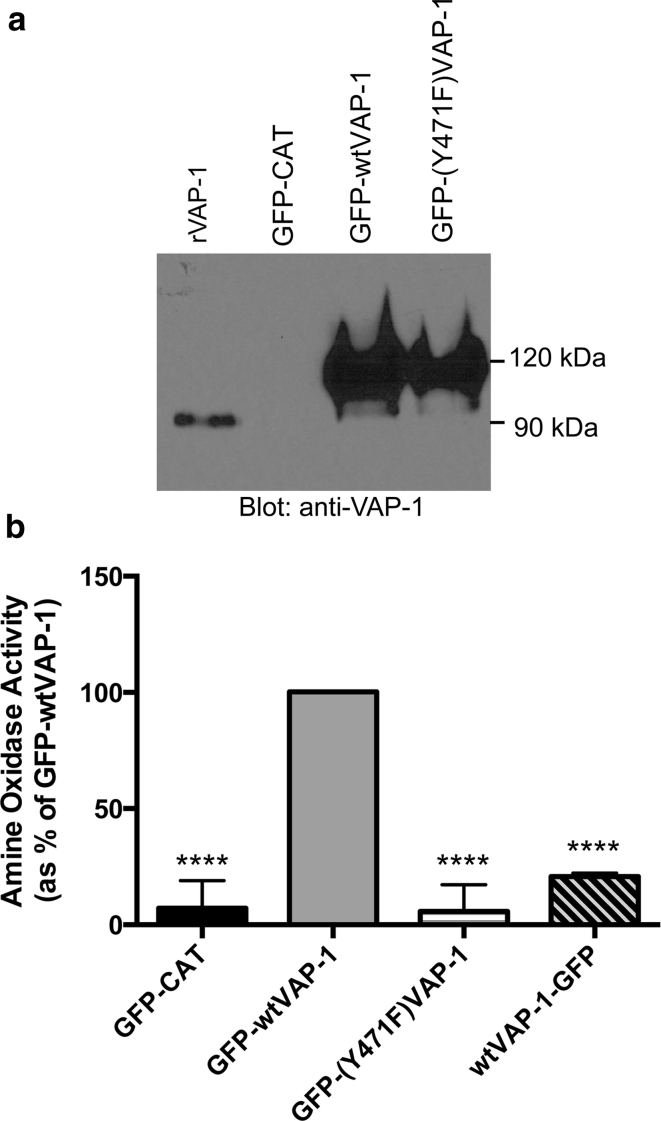
A GFP-wtVAP-1 fusion protein expressed in HEK293 was enzymatically active **a** GFP-wtVAP-1 and the enzyme dead mutant GFP-(Y471F)VAP-1 expressed in HEK293 had a molecular weight of approximately 115 kDa (predicted 113 kDa) as assessed by western blot (detection: anti-VAP-1 TK8-14 mAb). This was greater than the molecular weight of recombinant VAP-1 (rVAP-1, corresponding to the extracellular domain of VAP-1) due to the presence of the GFP moiety. There was no VAP-1 detectable in GFP-CAT transfected cells. **b** GFP-wtVAP-1 expressed in HEK293 was capable of deaminating the artificial substrate benzylamine as assessed by Amplex red assay. An enzyme-dead mutant GFP-(Y471F)VAP-1, the control protein GFP-CAT and a C-terminal GFP fusion protein, wtVAP-1-GFP exhibited only very low basal activity (****p < 0.001 *versus* GFP-wtVAP-1, one-way analysis of variance with Tukey’s post test, Graphpad Prism v.6.0a)

The expression of these constructs in HEK293 cells was confirmed by western blotting (Fig. 1a) with a transfection efficiency of >80 % as determined by flow cytometry (data not shown). The enzyme activity of GFP-wtVAP-1 was confirmed using benzylamine as a substrate (Fig. 1b). Both GFP-CAT and GFP-(Y471F)VAP-1 exhibited extremely low basal levels of activity. A C-terminal GFP-fusion protein, wtVAP-1-GFP, was also catalytically inactive, presumably as a result of blockade of the enzyme active site by the GFP moiety.

### GFP-VAP-1 localizes to vesicles in both hepatic endothelial and stromal cells

**Fig. 2:**
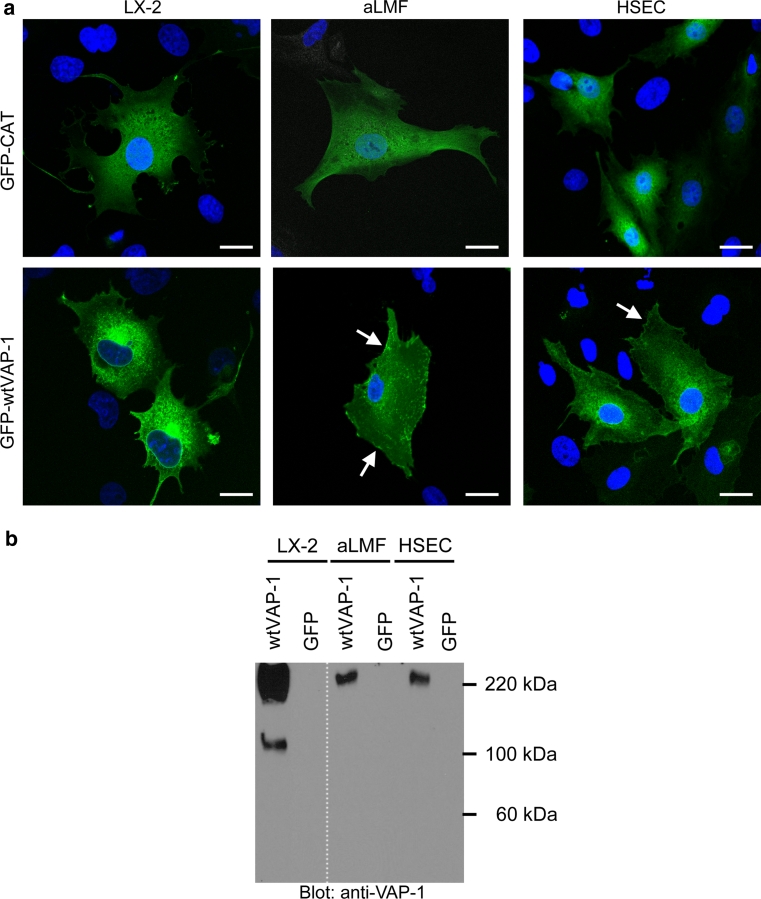
A GFP-VAP-1 fusion protein localized to vesicles in endothelial cells, fibroblasts and an hepatic stellate cell line **a** GFP-CAT (*top panels*) and GFP-wtVAP-1 (*lower panels*) fusion proteins produced by transient transfection of LX-2, aLMF and HSEC were imaged using multicolour confocal microscopy. Nuclei stained with DAPI are shown in *blue*. *Scale bar* 20 μm **b** Dimeric GFP-wtVAP-1 was detected in cell lysates of LX-2, aLMF and HSEC using the anti-VAP-1 antibody, TK8-14. Samples for aLMF and HSEC were loaded at 8 μg/lane and LX-2 at 40 μg/lane

To determine the localization of VAP-1 in HSEC, aLMF and LX-2, cells were transiently transfected with plasmid-encoding GFP-fusion proteins and visualized by confocal microscopy. We used Nucleofection-based technology to deliver the plasmid as the transfection efficiency was low with traditional lipid-based approaches. We routinely observed >50 % efficiency of transfection in LX-2 cells, but the primary cells showed more variability (30–70 % efficiency); in all cases, the viability following Nucleofection was high (>70 %, data not shown). Figure 2a demonstrates that in all cell types GFP-wtVAP-1 had a distinct perinuclear distribution and was concentrated in numerous vesicles and cell protrusions. Overall, there was little accumulation of GFP signal on the membrane of the stromal cells, whereas cell surface GFP staining was more evident in HSEC (arrows), reflecting the differences in role for these two cell types: endothelial VAP-1 is involved in recruitment of leukocytes from flow which would require surface-bound protein for capture, whereas stromal cells have little exposure to flow and might not require the protein to be accessible at the surface of the cell. There were no macroscopic differences in distribution of the GFP-wtVAP-1 and GFP-(Y471F)VAP-1 fusion proteins (Online resource: supplementary Fig. 1), and treatment of the cells with an amine oxidase substrate, benzylamine, or inhibitor, semicarbazide, had no effect on the localization of the proteins (Table 1, Online resource: supplementary Fig. 2). GFP-CAT was expressed throughout the cytoplasm in all three cell types studied.

**Table 1:** Effect of different compounds on the distribution of GFP-wtVAP-1 in different cell types

Compound	Action	LX-2	aLMF	HSEC
Semicarbazide	SSAO inhibitor	X	X	X
Methylamine	SSAO substrate	X	X	X
Brefeldin A	Golgi/endosome inhibitor	Majority of GFP signal retained in Golgi		
Monensin	Golgi inhibitor	X	X	X
Jasplakinolide	Actin inhibitor	X	Minor changes in distribution of GFP signal	
Cytochalasin D	Actin inhibitor	GFP and VAP-1 distribution largely unaffected relative to cell shape. Association with cell protrusions more pronounced		
Nocodzaole	Microtubule inhibitor	X	Minor changes in distribution of GFP signal	
Marimastat	Metalloproteinase inhibitor	X	X	X
Bafilomycin A1	H + -ATPase inhibitor	Induced co-localization of GFP and anti-VAP-1 signal		

“X” denotes no gross effect on distribution of GFP signal

Western blotting of the transfected cells for GFP-VAP-1 demonstrated that the protein was expressed in both aLMF and HSEC at similar levels (equal protein loading) at 240 kDa (Fig. 2b). Increased protein yields were obtained from transfection of the LX-2 cell line and analysis of this enriched cell lysate by western blotting confirmed that the dimeric form predominates over the monomer, despite the presence of reducing agent during sample preparation. There was no evidence for cleavage by proteinases in any of the samples despite stromal cells being a rich source of these enzymes. Furthermore, the addition of marimastat, a metalloproteinase inhibitor, had little effect on the subcellular distribution of the fusion proteins (Table 1).

### GFP-VAP-1 is associated with early endosomes and lyososomal trafficking

**Fig. 3:**
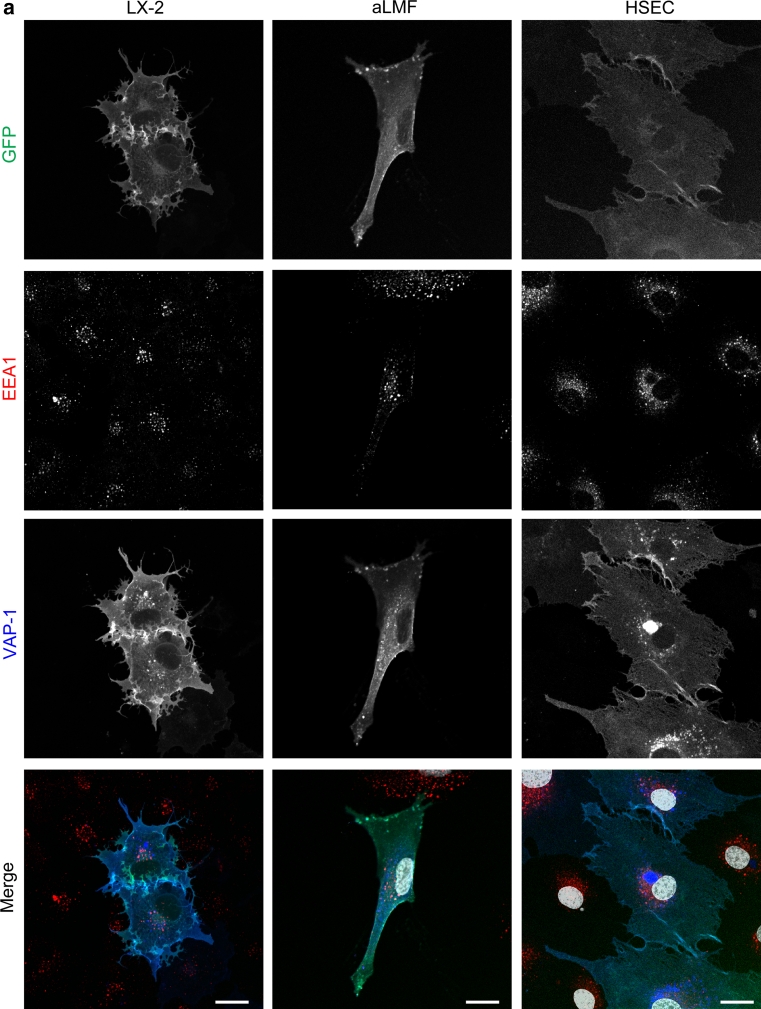

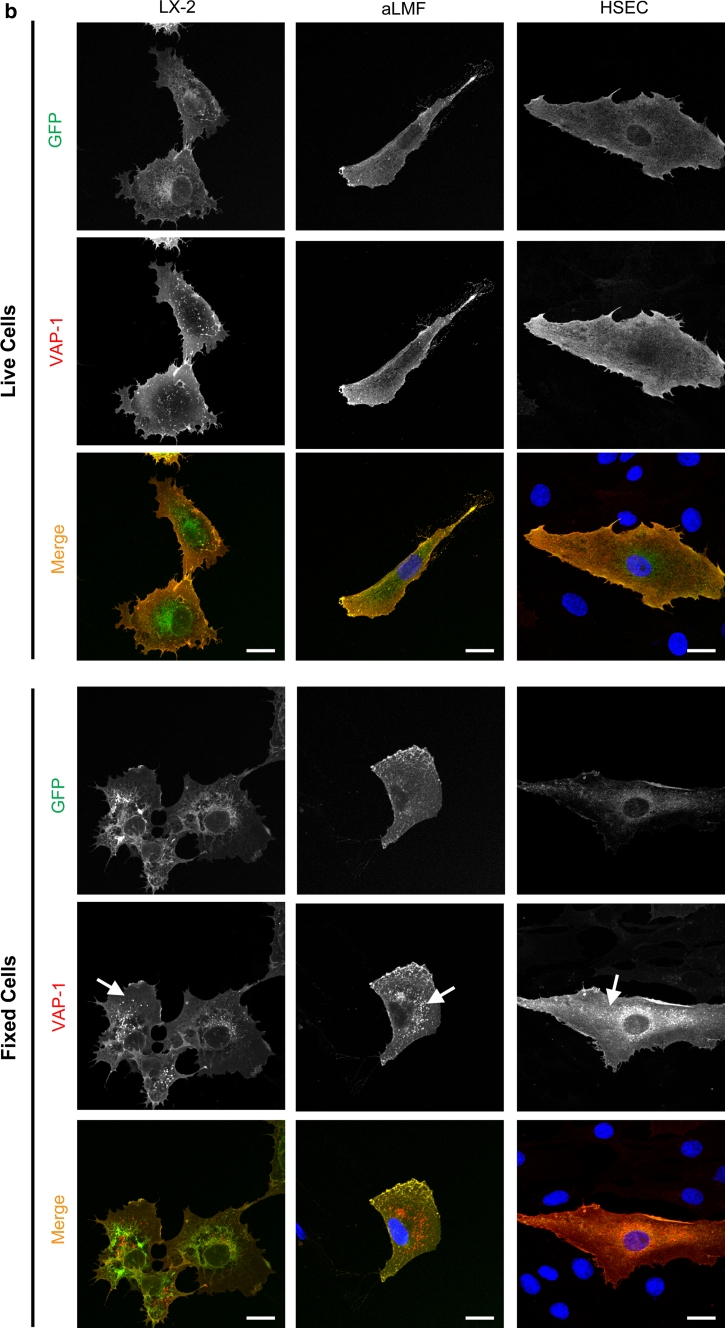
GFP-wtVAP-1 localizes to endosomes and other cellular vesicles. **a** Multicolour confocal microscopy indicated that GFP-wtVAP-1 co-localized with the early endosomal marker EEA-1. Merged images: GFP *green*; EEA-1 *red*; VAP-1 *blue*; nuclei *white*. **b** The distribution of GFP and anti-VAP-1 antibody fluorescence in live and fixed cells revealed the presence of intracellular GFP^low^VAP-1^high^ vesicles in fixed, but not live cells. Merged images: GFP *green*; VAP-1 *red*; nuclei *blue*. *Scale bar* 20 μm

Immunofluorescent staining of organelles within VAP-1 transfected cells was analysed by multicolour confocal microscopy and revealed that both the wild-type and enzyme-dead mutant of VAP-1 were expressed in endoplasmic reticulum (GRP94), Golgi (GM130) (Online resource: supplementary Fig. 3 a,b) and early endosomes (EEA1) (Fig. 3a). Although the majority of the GFP signal co-localized with staining for VAP-1 (as detected with anti-VAP-1 antibody), we observed some vesicles in both stromal and endothelial cells that were low/negative for GFP, but stained brightly for VAP-1. These vesicles were present in all fixed cells (arrows; Fig. 3b, lower panel, and Online resource: supplementary Fig. 4) but were not detected in cells treated with anti-VAP-1 antibody before fixation (Fig. 3b, upper panel). We did not observe any non-specific uptake of an isotype matched control in GFP-wtVAP-1 transfected cells or any similar staining pattern in those cells that had been transfected with GFP-CAT (data not shown). Staining of LX-2 cells with an antibody raised against GFP showed that while the intrinsic GFP fluorescence signal was diminished in these vesicles the GFP moiety had not been lost (co-localization with VAP-1 staining, Online resource: supplementary Fig. 5). This suggested that there might be a mechanism whereby the fluorescence of the GFP is quenched in these vesicles.

**Fig. 4:**
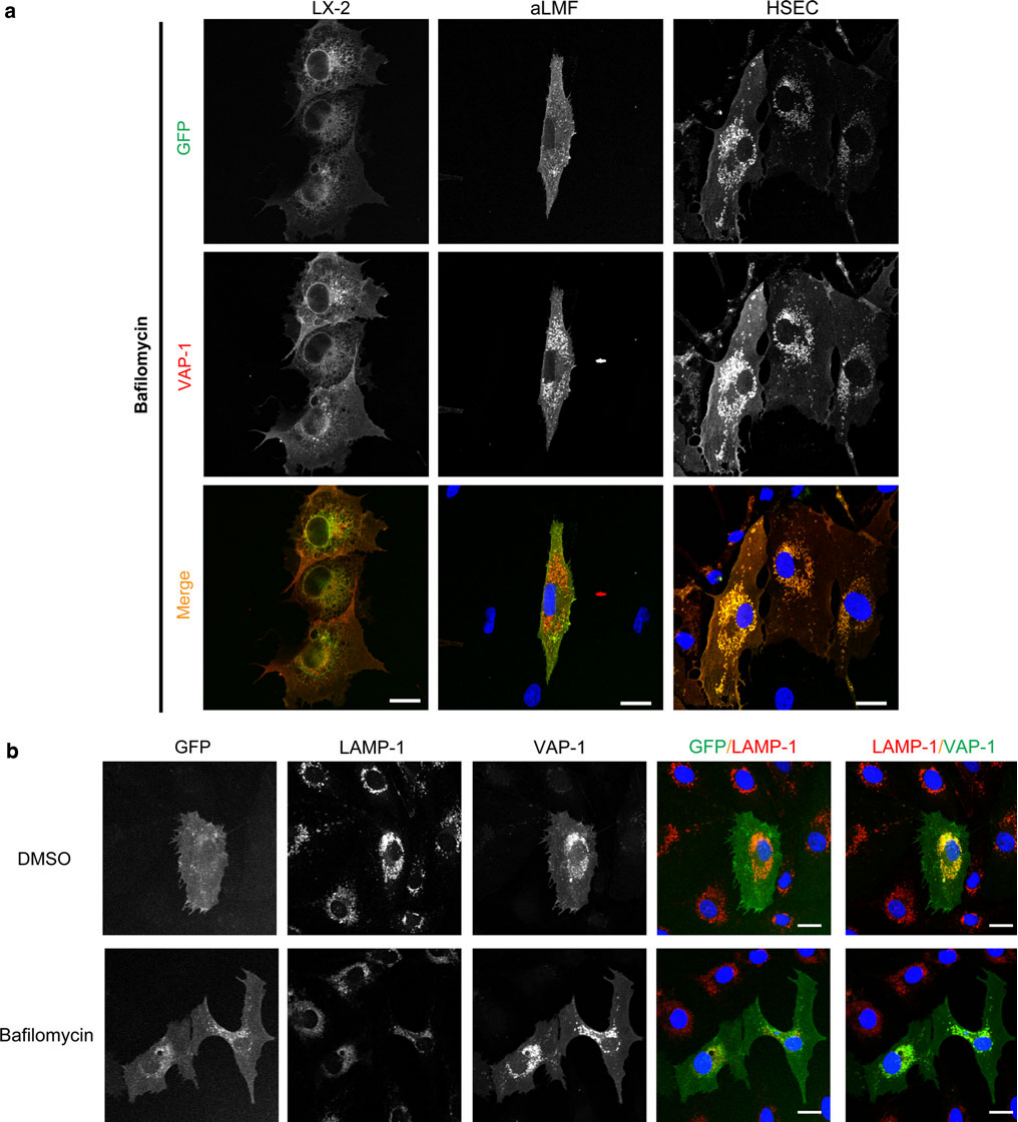

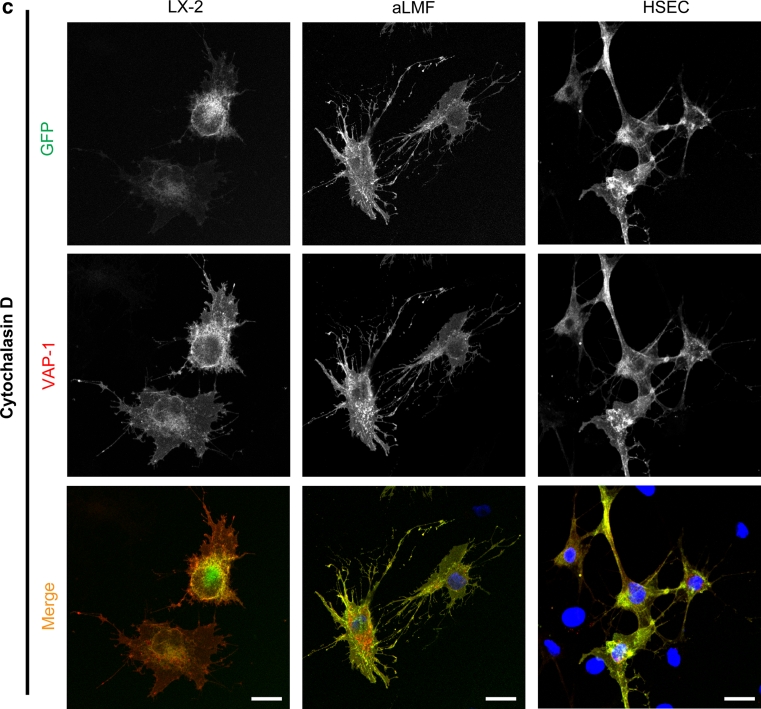
Treatment of cells with bafilomycin, and staining for LAMP-1 revealed that GFP-wtVAP-1 localizes within vesicles of low *p*H **a** In the presence of bafilomycin A1 (1 μM), the GFP and VAP-1 signals from GFP-wtVAP-1 showed greater co-localization when compared with vehicle treated controls. There was no difference in the distribution of GFP signal in GFP-CAT transfected cells (data not shown). Merged images: GFP *green*; VAP-1 *red*; nuclei *blue*. **b** Lysosomal-associated membrane protein-1 (LAMP-1) co-localized with anti-VAP-1 staining in both vehicle and bafilomycin A1 treated HSEC, whereas an overlap with the GFP signal was only seen in the bafilomycin A1 treated cells. Merged images: GFP/VAP-1 *green*; LAMP-1 *red*; nuclei *blue*. **c** Cytochalasin D (10 μM), a disruptor of the actin cytoskeleton, altered the distribution of VAP-1 with the greatest effect observed in HSEC. *Scale bar* 20 μm

Incubation of the cells with the protein transport inhibitor brefeldin A prevented the export of GFP-wtVAP-1 from the ER/Golgi, whereas monensin had very little effect (Table 1 and data not shown). It is known that brefeldin A can affect the formation of endosomes in addition to targeting the Golgi apparatus and this might explain the differences observed between these two inhibitors. The addition of bafilomycin, which inhibits vacuolar-type H+-ATPase driven acidification of endosomes and lysosomes (Bowman et al. 1988), resulted in an accumulation of GFP signal in vesicles that also stained positive for VAP-1 (Fig. 4a). Vehicle controls (DMSO or ethanol) at equivalent concentrations had no effect. Using LAMP-1 as a lysosomal marker we showed that in the absence of bafilomycin, VAP-1 could be detected in lysosomes via antibody binding, but the corresponding GFP signal was absent (Fig. 4b, top panels). However, upon the addition of bafilomycin the GFP signal overlapped with that of LAMP-1 and VAP-1 (Fig. 4b, lower panels) suggesting that the low pH of recycling vesicles quenches the GFP signal associated with GFP-wtVAP-1 (but does not disrupt the interaction with the anti-VAP-1 antibody), giving rise to GFP^low^ VAP-1^high^ vesicles (Figs. 3 , 4). Upon treatment with bafilomycin, the rise in pH in these vesicles due to inhibition of the H+ -ATPase establishes an environment in which GFP fluorescence is maintained, leading to complete co-localization of the GFP and VAP-1 fluorescent signals. This effect of bafilomycin was absent in cells expressing GFP-CAT (data not shown) suggesting that the redistribution was not due to a toxic effect of the compound.

The addition of cytochalasin D to disrupt the actin cytoskeleton altered the cell morphology and enhanced the association of both GFP and VAP-1 signals with cell projections (Fig. 4c). The effects of other inhibitors are summarised in Table 1. Supplementation of the media with jasplakinolide which stabilizes actin filaments had no detectable effect on the distribution of VAP-1, and destabilization of microtubules by nocodazole had little effect on the distribution of the GFP-proteins or total VAP-1 at the concentrations used in this study (data not shown).

## Discussion

We have shown that a fusion protein where a GFP moiety is linked to the N-terminus of VAP-1 can be expressed in endothelial and stromal cells and retain its catalytic activity. The fusion protein was monomeric under denaturing conditions in HEK293 whole cell lysates, whereas the dimeric form predominated in lysates prepared from transfected endothelial and stromal cells (Figs. 1, 2). The reasons for this are not yet clear as the lysates were prepared under the same conditions. The dimer interface is formed by opposing D4 domains of each of the VAP-1 monomers including an inter-subunit disulfide bond between opposing Cys748 residues (Airenne et al. 2005). Changes in glycosylation of the protein, the presence of previously undescribed binding partners or orientation of the transmembrane domain might protect the dimer from dissociation some cell types. It is unlikely that this effect was due to multimerization of the GFP moiety as we used EmGFP that has a low propensity to dimerize (Shaner et al. 2007).

GFP-wtVAP-1 showed some membranous staining, but was mainly distributed throughout the cytoplasm associated with subcellular vesicles via a mechanism that is independent of enzyme activity. These data are consistent with those obtained for endothelial and smooth muscle cell lines where untagged VAP-1 was shown to have both a cytosolic distribution and an association with flotillin-containing lipid rafts (Sole and Unzeta 2011). This may allow the protein to be mobilised rapidly during inflammation as has been demonstrated previously for endothelial cells (Salmi and Jalkanen 2001), possibly via association with the actin cytoskeleton as a treatment of the cells with cytochalasin D disrupted the trafficking of GFP-wtVAP-1.

A proportion of the VAP-1-positive vesicles were negative/weak for GFP. Our data suggest that GFP-VAP-1 fusion protein that is being degraded or subjected to reuptake at the membrane into vesicles experiences a low pH environment in which the fluorescence of the GFP moiety is quenched (pKa of EmGFP is approximately 6.0; the pH of lysosomes is around pH 4.5)(Patterson et al. 1997). This would be advantageous in live cell imaging techniques, where the trafficking of newly synthesised GFP-VAP-1 protein can be studied without the complication of protein that has been targeted for degradation.

## Electronic supplementary material

Below is the link to the electronic supplementary material.

Supplementary material 1 (PDF 41 kb)

Supplementary material 2 (TIFF 2738 kb)

Supplementary material 3 (TIFF 2114 kb)

Supplementary material 4 (TIFF 3718 kb)

Supplementary material 5 (TIFF 2823 kb)

Supplementary material 6 (TIFF 3040 kb)

Supplementary material 7 (TIFF 6626 kb)
