# Supplementary material for: Cellular localization and trafficking of vascular adhesion protein-1 as revealed by an N-terminal GFP fusion protein
Source: J Neural Transm (Vienna). 2013 Mar 9;120(6):951–61. doi: 10.1007/s00702-013-1003-3 (PMC3664183; doi:10.1007/s00702-013-1003-3)
Supplement: Supplementary file 1 — Supplementary material 1 (PDF 41 kb) [file 702_2013_1003_MOESM1_ESM.pdf]

Supplementary Figures for: Journal of Neural Transmission

Cellular localization and trafficking of Vascular Adhesion Protein-1 as revealed by an N-terminal GFP fusion protein

Chris J. Weston[1,2], Emma L. Shepherd[1] and David H. Adams[1]

[1]Centre for Liver Research and NIHR Biomedical Research Unit,

5th Floor Institute of Biomedical Research, MRC Centre for Immune Regulation,

College of Medicine and Dentistry,

University of Birmingham,

Edgbaston, Birmingham, UK.

B15 2TT

[2]Corresponding author

(Tel: +44 (0)121 415 8785, Fax: +44 (0)121 415 8701, Email:

c.j.weston@bham.ac.uk)

Supplementary Fig. 1 GFP-(Y471F)VAP-1 shows a subcellular distribution that is similar to that of GFP-wtVAP-1

Multicolour confocal microscopy of transfected aLMF indicated that GFP-(Y471F)VAP-1 localized to subcellular compartments (Golgi, GM-130; endoplasmic reticulum, GRP94; early endosomes, EEA1) with a distribution pattern that was similar to that of GFP-wtVAP-1. Merged image: GFP, green; organelles, red; VAP-1, blue; nuclei, white. Scale bar: 20 µm.

Supplementary Fig. 2 Treatment of LX-2 or aLMF with methylamine or semicarbazide had little effect on the distribution of the GFP or VAP-1 signal in GFP-wtVAP-1 transfected cells.

Transfected cells were treated for 30 minutes with either methylamine (1 mM) or semicarbazide (250 µM) and subsequently fixed. Merged images show the distribution of GFP signal (green), VAP-1 (red) and nuclei (aLMF only, white). Areas of co-localization are coloured yellow. There were no gross differences between the no treatment groups and those that received either methylamine or semicarbazide. Scale bar: 20 µm.

Supplementary Fig. 3 GFP-wtVAP-1 localizes to endoplasmic reticulum and Golgi apparatus

Multicolour confocal microscopy indicated that GFP-wtVAP-1 co-localized with the endoplasmic reticulum marker GRP94 (a) and the Golgi marker GM-130 (b) in LX-2, aLMF and HSEC. Merged images: GFP, green; GRP94/GM-130, red; VAP-1, blue; nuclei (aLMF and HSEC only), white. Scale bar: 20 µm.

Supplementary Fig. 4 GFP-wtVAP-1 has a perinuclear distribution in LX-2, aLMF and HSEC

Magnified images of LX-2, aLMF and HSEC transfected with GFP-wtVAP-1 demonstrated that both the GFP and VAP-1 signals show a perinuclear distribution with the most pronounced effects seen in LX-2 and HSEC. Scale bar: 20 μm.

Supplementary Fig. 5 Vesicles that demonstrated weak intrinsic GFP fluorescence showed strong immunoreactivity to an anti-GFP antibody

Multicolour confocal microscopy of GFP-wtVAP-1 transfected LX-2 cells demonstrated that some VAP-1-positive vesicles had low intrinsic GFP fluorescence (arrows, leftmost merged images; GFP green, VAP-1 red, co-localization shown in yellow/orange) despite showing immunoreactivity towards an anti-GFP antibody (rightmost merged images; anti-GFP green, VAP-1 red, co-localization shown in yellow/orange). Nuclei stained with DAPI are shown in blue. Scale bar: 20 μm.
