# Supplementary figures and images for: Cellular localization and trafficking of vascular adhesion protein-1 as revealed by an N-terminal GFP fusion protein
Source: J Neural Transm (Vienna). 2013 Mar 9;120(6):951–61. doi: 10.1007/s00702-013-1003-3 (PMC3664183; doi:10.1007/s00702-013-1003-3)

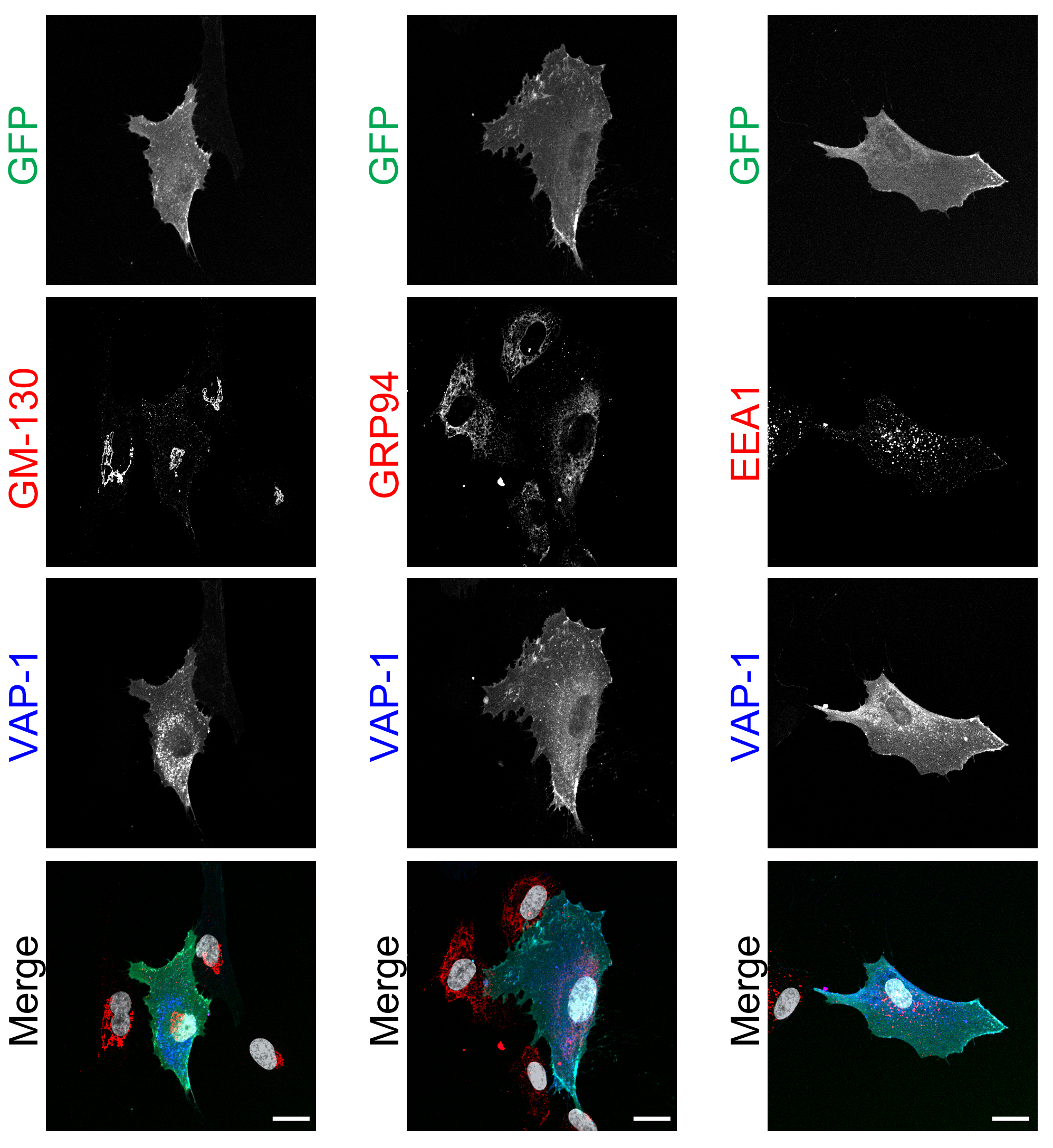

Supplement: Supplementary file 2 — Supplementary material 2 (TIFF 2738 kb) [file 702_2013_1003_MOESM2_ESM.tif]

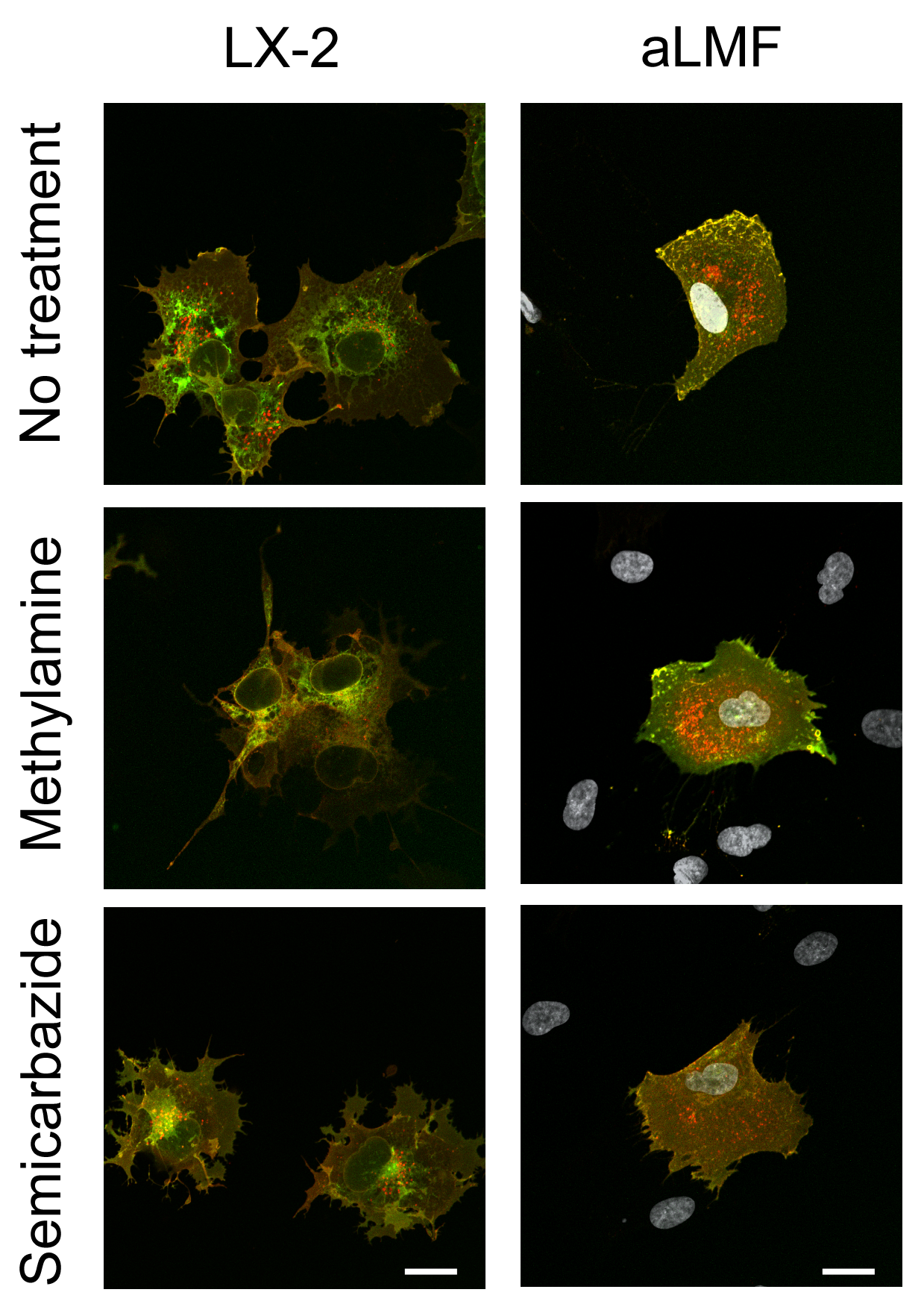

Supplement: Supplementary file 3 — Supplementary material 3 (TIFF 2114 kb) [file 702_2013_1003_MOESM3_ESM.tif]

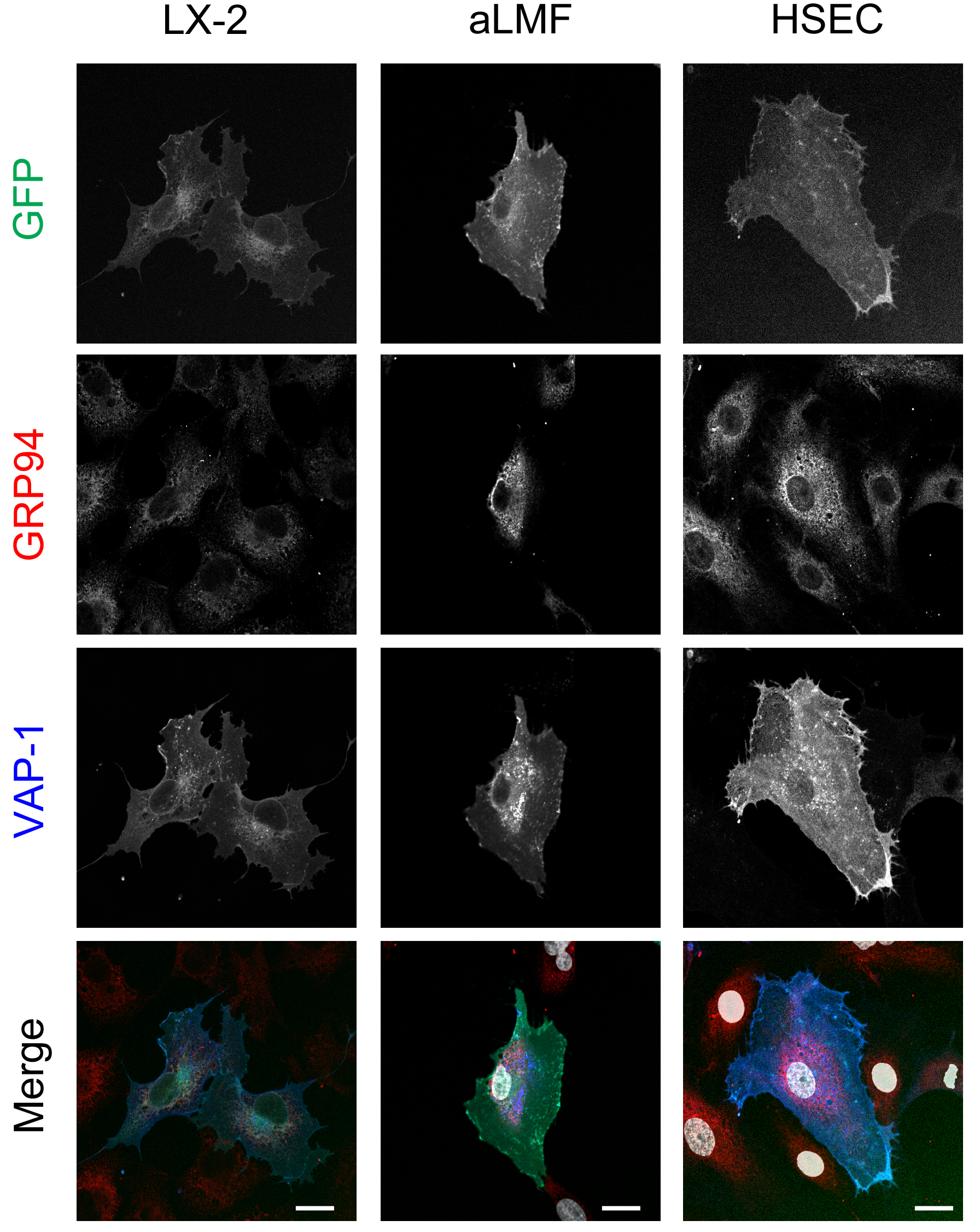

Supplement: Supplementary file 4 — Supplementary material 4 (TIFF 3718 kb) [file 702_2013_1003_MOESM4_ESM.tif]

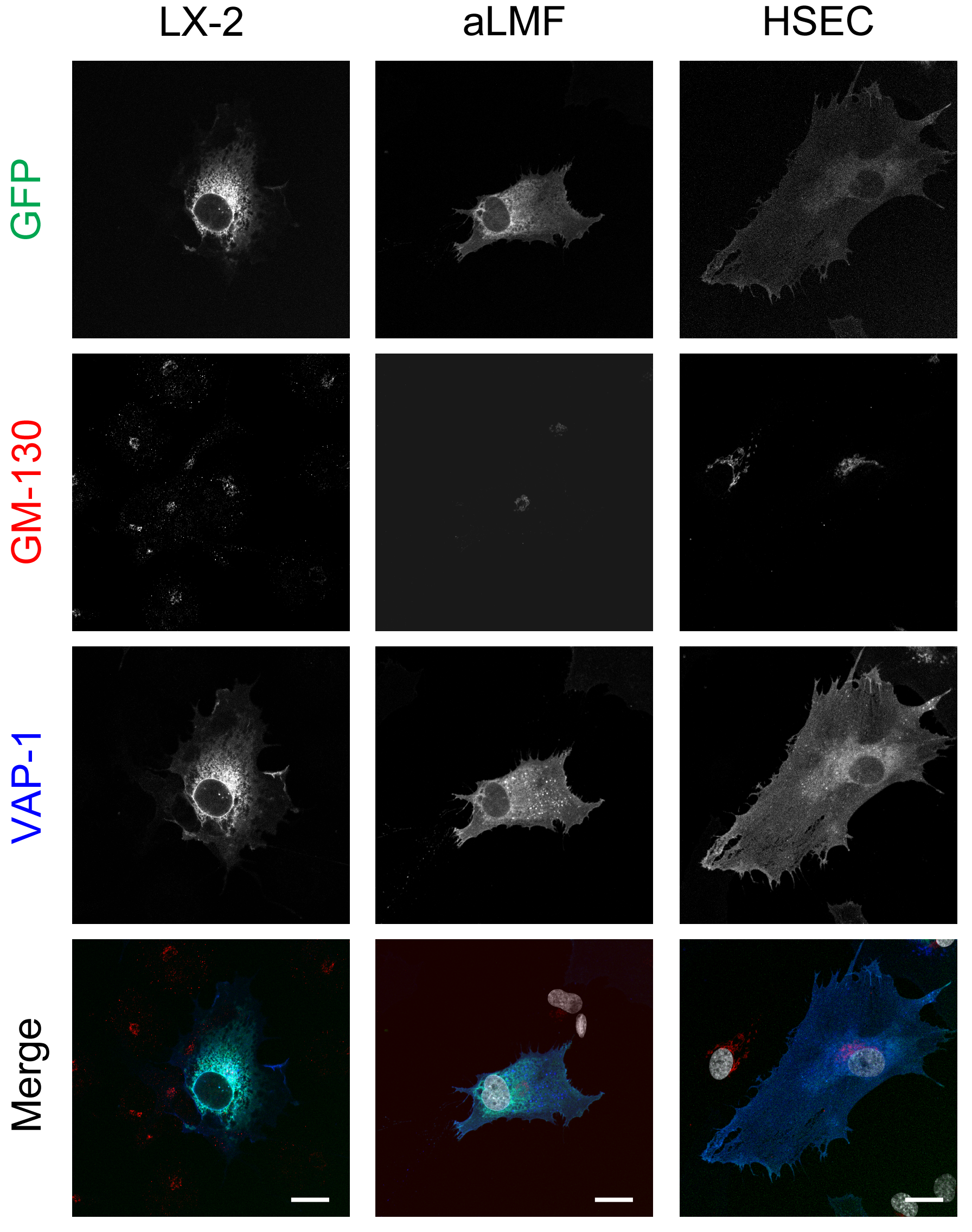

Supplement: Supplementary file 5 — Supplementary material 5 (TIFF 2823 kb) [file 702_2013_1003_MOESM5_ESM.tif]

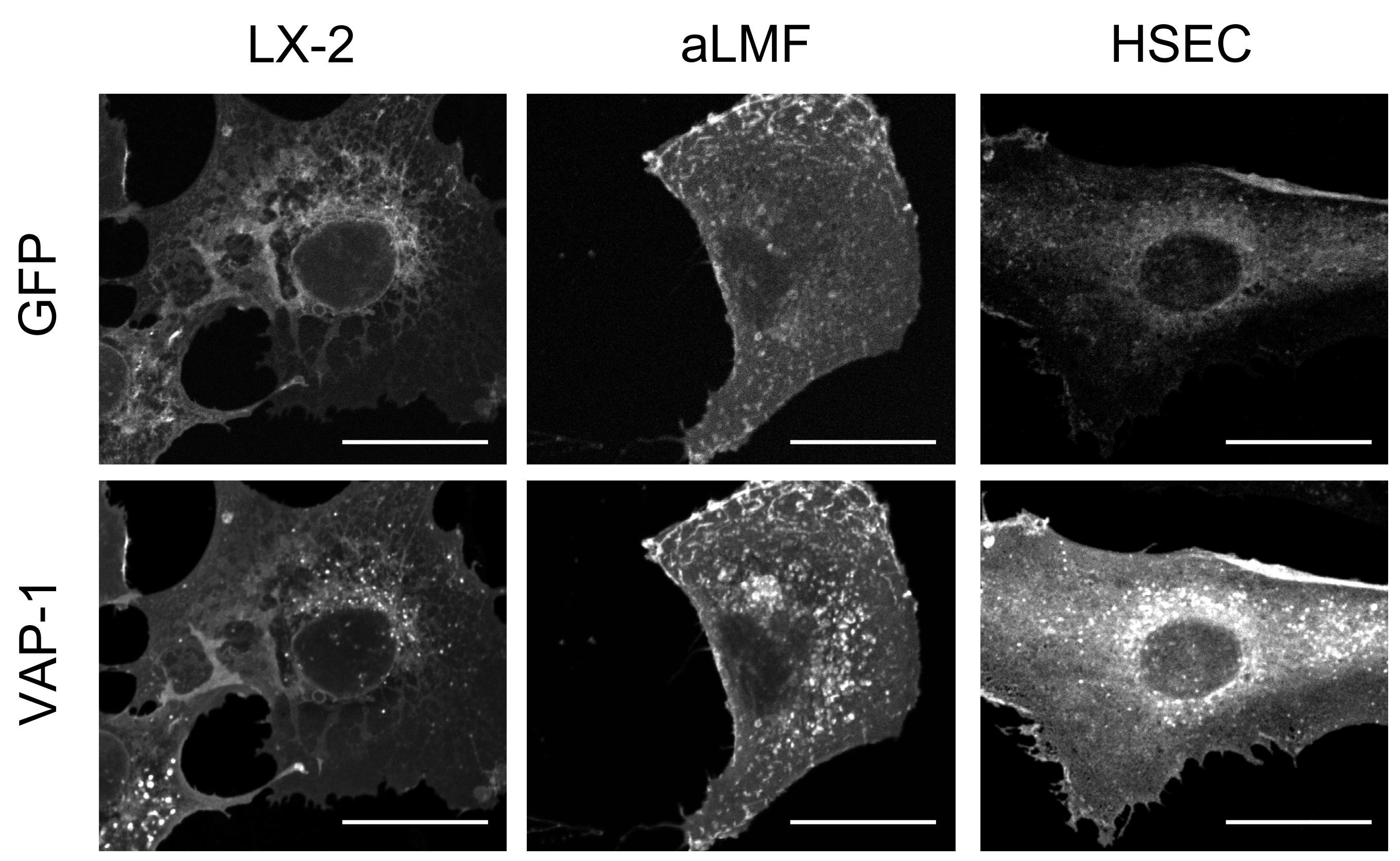

Supplement: Supplementary file 6 — Supplementary material 6 (TIFF 3040 kb) [file 702_2013_1003_MOESM6_ESM.tif]

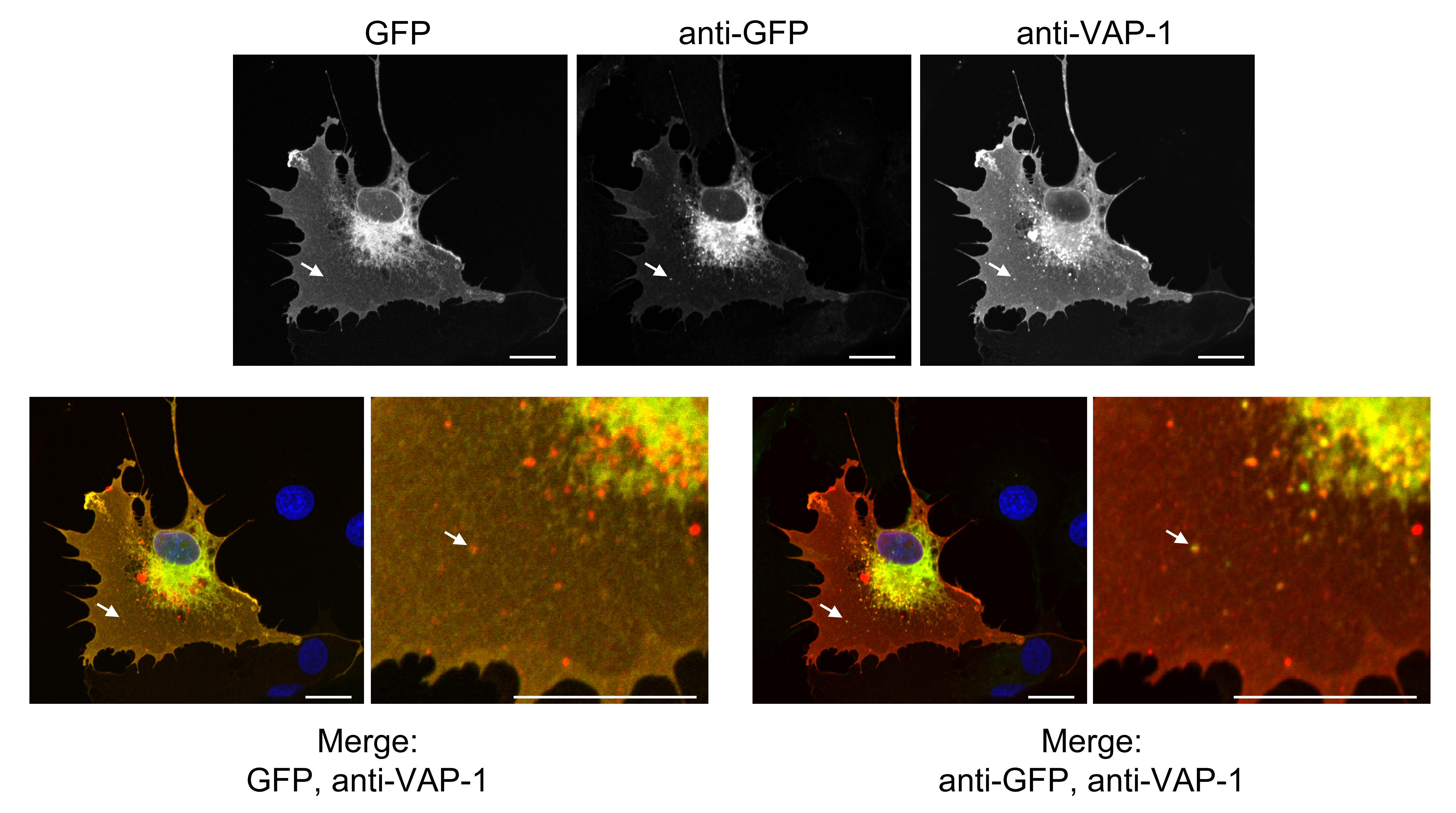

Supplement: Supplementary file 7 — Supplementary material 7 (TIFF 6626 kb) [file 702_2013_1003_MOESM7_ESM.tif]
